# Associations between schistosomiasis and HIV‐1 acquisition risk in four prospective cohorts: a nested case‐control analysis

**DOI:** 10.1002/jia2.25534

**Published:** 2020-06-25

**Authors:** Aaron F Bochner, Jared M Baeten, W Evan Secor, Govert J van Dam, Adam A Szpiro, Sammy M Njenga, Paul L A M Corstjens, Austin Newsam, Nelly R Mugo, Connie Celum, Andrew Mujugira, R Scott McClelland, Ruanne V Barnabas

**Affiliations:** ^1^ Department of Epidemiology University of Washington Seattle WA USA; ^2^ Department of Global Health University of Washington Seattle WA USA; ^3^ School of Medicine University of Washington Seattle WA USA; ^4^ Division of Parasitic Diseases and Malaria Center for Global Health Centers for Disease Control and Prevention Atlanta GA USA; ^5^ Department of Parasitology Leiden University Medical Center Leiden the Netherlands; ^6^ Department of Biostatistics University of Washington Seattle WA USA; ^7^ Kenya Medical Research Institute Nairobi Kenya; ^8^ Department of Cell and Chemical Biology Leiden University Medical Center Leiden the Netherlands; ^9^ Infectious Diseases Institute College of Health Sciences Makerere University Kampala Uganda

**Keywords:** HIV epidemiology, coinfections, risk factors, Africa, *Schistosoma mansoni*, *Schistosoma haematobium*

## Abstract

**Introduction:**

Globally, schistosomes infect approximately 200 million people, with 90% of infections in sub‐Saharan Africa. Schistosomiasis is hypothesized to increase HIV‐1 acquisition risk, and multiple cross‐sectional studies reported strong associations. We evaluated this hypothesis within four large prospective cohorts.

**Methods:**

We conducted nested case‐control analyses within three longitudinal cohorts of heterosexual HIV‐1 serodiscordant couples and one female sex worker (FSW) cohort from Kenya and Uganda. The serodiscordant couples studies were conducted between 2004 and 2012 while the FSW cohort analysis included participant follow‐up from 1993 to 2014. Cases HIV‐1 seroconverted during prospective follow‐up; three controls were selected per case. The presence of circulating anodic antigen in archived serum, collected prior to HIV‐1 seroconversion, identified participants with active schistosomiasis; immunoblots determined the schistosome species. Data from serodiscordant couples cohorts were pooled, while the FSW cohort was analysed separately to permit appropriate confounder adjustment.

**Results:**

We included 245 HIV‐1 seroconverters and 713 controls from the serodiscordant couples cohorts and 330 HIV‐1 seroconverters and 962 controls from the FSW cohort. The prevalence of active schistosomiasis was 20% among serodiscordant couples and 22% among FSWs. We found no association between schistosomiasis and HIV‐1 acquisition risk among males (adjusted odds ratio (aOR) = 0.99, 95% CI 0.59 to 1.67) or females (aOR = 1.21, 95% CI 0.64 to 2.30) in serodiscordant couples. Similarly, in the FSW cohort we detected no association (adjusted incidence rate ratio (aIRR) = 1.11, 95% CI 0.83 to 1.50). Exploring schistosome species‐specific effects, there was no statistically significant association between HIV‐1 acquisition risk and *Schistosoma mansoni* (serodiscordant couples: aOR = 0.90, 95% CI 0.56 to 1.44; FSW: aIRR = 0.83, 95% CI 0.53 to 1.20) or *Schistosoma haematobium* (serodiscordant couples: aOR = 1.06, 95% CI 0.46 to 2.40; FSW: aIRR = 1.64, 95% CI 0.93 to 2.87) infection.

**Conclusions:**

Schistosomiasis was not a strong risk factor for HIV‐1 acquisition in these four prospective studies. *S. mansoni* was responsible for the majority of schistosomiasis in these cohorts, and our results do not support the hypothesis that *S. mansoni* infection is associated with increased HIV‐1 acquisition risk. *S. haematobium* infection was associated with a point estimate of elevated HIV‐1 risk in the FSW cohort that was not statistically significant, and there was no trend towards a positive association in the serodiscordant couples cohorts.

## 1 Introduction

Schistosomiasis, a parasitic disease caused by the schistosome flatworm, affects approximately 200 million people globally [1], over 90% of whom live in sub‐Saharan Africa [2, 3]. In Africa, schistosomiasis is predominantly caused by two schistosome species: *Schistosoma haematobium* and *Schistosoma mansoni* [4]. Several cross‐sectional studies found strong positive associations between prevalent *S. haematobium* or *S. mansoni* infection and HIV‐1 [5, 6, 7, 8], which supported the hypothesis that schistosomiasis increases HIV‐1 acquisition risk. However, findings from more recent studies are mixed [9, 10, 11, 12, 13]. Thus, there remains a need to validate this association in well‐powered longitudinal analyses.

Several biological mechanisms have been proposed to explain how schistosomiasis increases susceptibility to HIV‐1. Adult female schistosome worms lay hundreds of eggs daily into the venules in which they reside [14, 15], and eggs become deposited into host genital organs. *S. haematobium* and *S. mansoni* have differing pathologies, and eggs are most frequently observed in genital organs for women infected with *S. haematobium* [16]. These ova can remain trapped in genital mucosal tissues, causing an influx of immune cells, including CD4^+^ T‐cells that may be targeted by the HIV‐1 virus [17]. Trapped ova also induce neovascularization, resulting in mucosal fragility that may enable HIV‐1 to gain access into the bloodstream [18, 19]. Additionally, individuals with *S. mansoni* have higher concentrations of HIV‐1 co‐receptors CCR5 and CXCR4 on CD4^+^ T cells, which could increase susceptibility to HIV‐1 [20, 21]. Helminths including schistosomes have been shown to induce elevated levels of immune activation [22], which has been hypothesized to make individuals more susceptible to HIV infection [23, 24, 25]. Lastly, a recent study found *S. mansoni* infection associated with increased expression of α4β7 on blood CD4^+^ T cells [26], and increased pre‐HIV infection expression of α4β7 has been associated with increased rates of HIV acquisition among women [27].

In this analysis, we utilized data from four longitudinal studies conducted in Kenya and Uganda. Three of these cohorts enrolled HIV‐1 serodiscordant couples while one enrolled female sex workers (FSW). Our objective was to evaluate the hypothesis that schistosomiasis increases HIV‐1 acquisition risk.

## 2 Methods

### 2.1 Study population

Longitudinal data from four prospective cohort studies conducted in schistosomiasis endemic areas were included in this nested case‐control analysis. Three cohorts enrolled heterosexual HIV‐1 serodiscordant couples: The Partners in Prevention HSV/HIV Transmission Study [28], the Couples Observational Study [29], and the Partners Pre‐Exposure Prophylaxis (PrEP) Study [30]. These studies were conducted between 2004 and 2012 and enrolled more than 8500 couples for a duration of between 12 and 36 months. HIV‐negative partners were tested for HIV‐1 monthly or quarterly. The fourth cohort, the Mombasa Cohort, enrolled FSWs in Mombasa, Kenya. This prospective cohort enrolled 3471 women between 1993 and 2014. Enrolment was ongoing and women could maintain participation in the cohort for as long as they resided in Mombasa. Participants were invited to monthly clinic visits for HIV‐1 testing; detailed study procedures have been published elsewhere [31, 32, 33].

Inclusion criteria for HIV‐1 seroconverters (cases) in this analysis was consistent across all four cohorts. All participants aged 16 and above who were HIV‐1 seronegative at study enrolment, HIV‐1 seroconverted during study follow‐up, and had a serum or plasma sample collected prior to the HIV‐1 seroconversion study visit were included. Additionally, inclusion from the Couples Observational Study and Partners in Prevention HSV/HIV Transmission Study was restricted to participants enrolled at sites in Kenya and Uganda, where schistosomiasis is endemic [3]. Serodiscordant couples included in the analysis were enrolled at four sites in Kenya (Kisumu, Nairobi, Eldoret and Thika) and five sites in Uganda (Kampala, Tororo, Mbale, Kabwohe and Jinja) while all FSW were enrolled in Mombasa, Kenya.

Three controls were selected per HIV‐1 seroconverter, with appropriate sampling methodology used for the serodiscordant couples and FSW cohorts. Since the serodiscordant couples cohorts were all of relatively short duration, with little loss to follow‐up, controls were frequency‐matched to cases based on study, sex and study randomization arm (Partners PrEP only). This approach was used in previous nested case‐control analyses [34, 35]. Once control participants were sampled, a study visit was selected for each control for schistosomiasis testing and assessment of time‐varying confounders. For controls, this visit selection was frequency‐matched to the timing of the case samples selected for schistosomiasis testing using one‐year time bands, ensuring that cases and controls had the same distribution of timing of schistosomiasis testing. For the FSW cohort, which by design had variable lengths of participant follow‐up, controls were selected using incidence density sampling [36], matching on two‐year periods of study enrolment. Incidence density sampling has the advantage of reducing potential bias caused by differential loss to follow‐up between schistosomiasis infected and uninfected controls since the sampling probability is proportional to the amount of cohort follow‐up time accrued [37]. None of the study protocols for the four cohorts included screening or treatment for schistosomiasis.

All study protocols, including planned analyses for HIV‐1 transmission risk factors, were approved by the University of Washington Human Subjects Division as well as ethics review committees at each study site. The study was also reviewed by the U.S. Centers for Disease Control and Prevention (CDC), which deemed CDC personnel to be non‐engaged as they had no contact with study participants or access to personal identifiers. All study participants provided written informed consent.

### 2.2 Laboratory testing

Schistosomiasis testing was conducted using a three‐stage testing algorithm. First, all samples were tested by ELISA using soluble egg antigen (SEA) to detect antischistosomal antibodies [38]. Because the SEA ELISA cannot differentiate between active or resolved infection, SEA‐positive samples were tested for the presence of schistosome circulating anodic antigen (CAA) using the SCAA20 assay (detection threshold of 10 pg/mL) [39], which specifically detects active infections [40]. For samples that were SEA and CAA positive, species‐specific immunoblots were performed for *S. mansoni* and *S. haematobium* to identify the schistosome species causing infection [41]. Because schistosome antigen levels correlate with worm burden [39], results from the SCAA20 assay were used to classify infection intensity as low (10 to 99 pg/mL), medium (100 to 999 pg/mL), or high burden (≥1000 pg/mL), as done by others [42]. This differs from the traditional WHO classification of infection intensity based on median egg counts in faecal or urine samples, which were not available for these cohorts [43]. SEA and species‐specific immunoblot testing was performed by the CDC, while CAA testing was performed at Leiden University Medical Center.

HIV‐negative study participants were tested for HIV‐1 during each routine study visit. For the serodiscordant couples cohorts, dual rapid HIV‐1 antibody tests were performed during clinic visits, with confirmatory HIV‐1 enzyme immunoassay and western blot. For the FSW cohort, HIV‐1 testing was performed via an ELISA, with positive results confirmed by a second ELISA.

### 2.3 Statistical methods

For all cohorts, bivariate and multivariable models were used to assess associations between schistosomiasis and HIV‐1 acquisition risk. Data for the three serodiscordant couples cohorts were pooled and analysed together, while the FSW cohort was analysed in separate statistical models. The serodiscordant couples and FSW cohorts were analysed separately to permit adjustment for the complete set of possible confounders collected for each population and because different sampling methodologies were used. For the frequency‐matched cases and controls of the serodiscordant couples cohorts, logistic regression models were used to estimate odds ratios. Conditional logistic regression models were used for the FSW cohort’s incidence‐density matched cases and control to estimate incidence rate ratios. All analyses were done using robust standard errors and were performed using Stata version 13.1 (Stata Corporation, College Station, TX, USA).

For all cohorts, two types of potential confounders were identified prior to the analysis: *a priori* confounders identified through the available literature whose inclusion in all statistical models was pre‐determined, as well as a list of potential covariates only to be included if empirically found to be meaningful confounders (>10% change in the effect estimate, as done previously [29, 44, 45]). The lists of confounders and their definitions differed between the two populations, as the standard approach to covariate adjustment was used for each population. *A priori* confounders were age, sex and study/site combination for the serodiscordant couples cohorts and age, year of study enrolment and workplace (a marker of socio‐economic status) for the FSW cohort. Potential confounders were generally factors associated with HIV‐1 acquisition risk but whose association with schistosomiasis was inconsistent or unknown. For the serodiscordant couples cohorts, the potential confounders we evaluated were income, education, diagnosis of trichomoniasis, gonorrhoea or chlamydia, HSV‐2 serostatus, male circumcision status, PrEP study arm, contraceptive use (time‐varying), pregnancy status (time‐varying), unprotected sex (time‐varying), other sex partners (time‐varying) and genital ulcer disease (time‐varying). For the FSW cohort, potential confounders included education, parity, nationality (Kenyan/other), marital status, vaginal washing practices, unprotected sex (time‐varying), number of sex partners (time‐varying), contraceptive use (time‐varying), gonorrhoea (time‐varying), trichomoniasis (time‐varying) and HSV‐2 serostatus (time‐varying). None of these additional variables meaningfully (>10%) changed the effect estimates for our primary model for either the serodiscordant couples or FSW cohorts, and thus were not included in multivariable models.

We used analogous statistical models with the same sets of covariates to perform subgroup and sensitivity analyses. In the serodiscordant couples cohort, we performed subgroup analyses by sex to evaluate the hypothesis that the association between schistosomiasis and HIV‐1 acquisition is specific to females. Additionally, we performed sensitivity analyses evaluating associations by schistosome infection intensity and schistosome species. Infection intensity was modelled using indicator variables for each level of infection. Additionally, a test for trend was performed modelling infection intensity levels linearly and excluding individuals with past but not current infection. Schistosome species were modelled using indicator variables for *S. mansoni*, *S. haematobium* and infections caused by an undetermined species (CAA positive but tested negative for both species), which may represent recently acquired schistosome infections.

### 2.4 Role of the funding source

The funders had no role in study design, data collection and analysis, interpretation of data, decision to publish or preparation of the manuscript. The corresponding author had full access to all the data in the study and had final responsibility for the decision to submit for publication.

## 3 Results

### 3.1 Descriptive statistics of the study population

From the 7026 couples enrolled in the serodiscordant couples cohorts at sites in Kenya and Uganda, we identified 245 individuals who HIV‐1 seroconverted (94 from Partners in Prevention HSV/HIV Transmission Study, 13 from Couples Observational Study and 138 from Partners PrEP) and 713 frequency‐matched controls. In the Mombasa FSW Cohort, from 2160 HIV‐1 uninfected participants who enrolled in the cohort, 332 individuals seroconverted, of whom 330 had a blood sample available for schistosomiasis testing and were included in the analysis. A total of 990 control samples were selected, of whom 28 were later excluded due to insufficient sample volume available for schistosomiasis testing, leaving 962 controls included in the analysis. Characteristics of individuals who HIV‐1 seroconverted and controls are shown in Table 1. Among HIV‐1 seroconverters, samples tested for schistosomiasis were collected a median of 84 days (interquartile range (IQR), 53 to 92) and 166 days (IQR, 96 to 424) prior to the seroconversion study visit for individuals in the serodiscordant couple and FSW cohorts respectively.

**Table 1:** Participant characteristics

	Serodiscordant couples cohorts		FSW cohort	
	HIV seroconverted (N = 245)	Controls (N = 713)	HIV seroconverted (N = 330)	Controls (N = 962)
Age^a^				
16 to 24	52 (21%)	91 (13%)	72 (22%)	205 (21%)
25 to 34	120 (50%)	327 (46%)	176 (53%)	453 (47%)
≥35	73 (30%)	295 (41%)	82 (25%)	304 (32%)
Sex				
Female	128 (52%)	266 (37%)	330 (100%)	962 (100%)
Male	117 (48%)	447 (63%)	–	–
Education^b^				
<9 years	167 (68%)	437 (61%)	212 (64%)	607 (63%)
≥9 years	78 (32%)	276 (39%)	118 (36%)	355 (37%)
Married^c^				
Yes	238 (97%)	695 (97%)	176 (53%)	512 (53%)
No	7 (3%)	18 (3%)	154 (47%)	450 (47%)
Enrolment locationd				
Kenya	122 (50%)	381 (53%)	330 (100%)	962 (100%)
Uganda	123 (50%)	332 (47%)	–	–
Any unprotected sex^e^				
Yes	72 (30%)	139 (20%)	162 (49%)	447 (46%)
No	172 (70%)	572 (80%)	168 (51%)	515 (54%)
Number of sex partners^e^				
≤1	219 (91%)	626 (90%)	259 (78%)	729 (76%)
>1	22 (9%)	72 (10%)	71 (22%)	233 (24%)
Sexually transmitted infections^f^				
Yes	27 (11%)	57 (8%)	56 (17%)	77 (9%)
No	218 (89%)	656 (92%)	270 (83%)	769 (91%)
Contraceptive use, females only				
None	81 (63%)	153 (58%)	176 (53%)	629 (65%)
IUD/surgical	4 (3%)	17 (6%)	9 (3%)	49 (5%)
Implant/injectable	34 (27%)	75 (28%)	102 (31%)	192 (20%)
Oral contraceptive	9 (7%)	21 (8%)	43 (13%)	92 (10%)
Serodiscordant couples cohort				
Partners HSV/HIV transmission study	94 (38%)	262 (37%)	–	–
Couples observational study	13 (5%)	39 (5%)	–	–
Partners PrEP study	138 (56%)	412 (58%)	–	–
Workplace				
Nightclub	–	–	41 (12%)	249 (26%)
Bar/other	–	–	289 (88%)	713 (74%)

FSW, female sex worker; IUD, intrauterine device; PrEP, pre‐exposure prophylaxis.

^a^ For the serodiscordant couples cohorts, age at enrolment was assessed since the longest study duration was three years. For the FSW cohort, age was time‐varying

^b^ years of education at time of cohort enrolment

^c^ for the serodiscordant couples cohorts, marital status at the time of study enrolment was assessed. For the FSW cohort, marital status at enrolment was categorized as ever married vs. never married because few participants were married (18/1292)

^d^ for the serodiscordant couples cohorts, cases and controls were enrolled at four sites in Kenya [Kisumu (n = 192), Nairobi (n = 119), Eldoret (n = 100), and Thika (n = 92)) and five sites in Uganda (Kampala (n = 210), Tororo (n = 79), Mbale (n = 71), Kabwohe (n = 59) and Jinja (n = 36)]. Enrolment for the FSW cohort was done in Mombasa, Kenya

^e^ for the serodiscordant couples cohorts, sexual behaviours were assessed over the prior month. Some individuals had missing values for unprotected sex (n = 3) and number of sexual partners (n = 19). For the FSW cohort, average sexual behaviours were calculated for each year of cohort follow‐up. For both cohorts, sexual behaviours were assessed at all study visits and was time‐varying

^f^ for the serodiscordant couples cohorts, testing for sexually transmitted infections (trichomoniasis, gonorrhoea and chlamydia) was done at enrolment. For the FSW cohort, sexually transmitted infection testing (trichomoniasis and gonorrhoea) occurred at each study visit and was time‐varying, and some individuals lacked test results (n = 116).

In the serodiscordant couples cohorts, 32% (305/958) of samples were antischistosomal (SEA) antibody positive, of whom 64% (194/305) tested positive for schistosome antigens (CAA) indicating an active schistosome infection. In the FSW cohort, 34% (439/1292) of samples were antischistosomal antibody positive, of whom 66% (290/439) tested antigen positive. Thus, the prevalence of active schistosomiasis was 20% (194/958) within the serodiscordant couples cohorts and 22% (290/1292) within the FSW cohort. Additionally, among a 10% sample of antischistosomal antibody negative specimens from all cohorts, only 3% (4/142) tested antigen positive, indicating that the antibody assay was highly sensitive in these populations.

### 3.2 Schistosome infection and HIV‐1 acquisition risk

In the serodiscordant couples cohorts, we found no evidence of an association between schistosomiasis and HIV‐1 acquisition (Table 2). We estimated the association for all participants (adjusted odds ratio (aOR) = 1.08, 95% confidence interval (CI) 0.73 to 1.60) and separately for males (aOR = 0.99, 95% CI 0.59 to 1.67) and females (aOR = 1.21, 95% CI 0.64 to 2.30). In the FSW cohorts, we also found no evidence of an association between schistosomiasis and HIV‐1 acquisition (adjusted incidence rate ratio (aIRR) = 1.11, 95% CI 0.83 to 1.50). Because some mucosal damage from schistosome ova has been shown to persist beyond the period of active infection [46, 47], we also evaluated if ever having experienced a schistosome infection increased HIV‐1 acquisition risk (using the anti‐SEA antibody result), but found no association (Table 2).

**Table 2:** Associations between schistosomiasis and the risk of HIV‐1 acquisition

Serodiscordant couples cohorts	Bivariate				Multivariable model			
	HIV SC/Total (%)	OR	95% CI	*p*	aOR^a^	95% CI	*p*
Active schistosome infection							
All participants							
Noschistosomiasis	193/764 (25)	Ref	–	–	Ref	–	–
Schistosomiasis^b^	52/194 (27)	1.08	0.76 to 1.55	0.660	1.08	0.73 to 1.60	0.700
Males							
No schistosomiasis	86/425 (20)	Ref	–	–	Ref	–	–
Schistosomiasis^b^	31/139 (22)	1.13	0.71 to 1.80	0.602	0.99	0.59 to 1.67	0.981
Females							
No schistosomiasis	107/339 (32)	Ref	–	–	Ref	–	–
Schistosomiasis^b^	21/55 (38)	1.34	0.74 to 2.42	0.333	1.21	0.64 to 2.30	0.552

FSW cohort	HIV SC/Total (%)	IRR	95% CI	*p*	aIRR^c^	95% CI	*p*
Active schistosome infection							
Females							
No schistosomiasis	248/1002 (25)	Ref	–	–	Ref	–	–
Schistosomiasis^b^	82/290 (28)	1.20	0.89 to 1.61	0.224	1.11	0.83 to 1.50	0.478

Serodiscordant couples cohorts	Bivariate				Multivariable model			
	HIV SC/total (%)	OR	95% CI	*p*	aOR^a^	95% CI	*p*
Active or prior schistosome infection							
All participants							
Antibody (anti‐SEA) negative	172/653 (26)	Ref	–	–	Ref	–	–
Antibody (anti‐SEA) positive	73/305 (24)	0.88	0.64 to 1.21	0.427	0.87	0.62 to 1.24	0.444
Males							
Antibody (anti‐SEA) negative	77/355 (22)	Ref	–	–	Ref	–	–
Antibody (anti‐SEA) positive	40/209 (19)	0.85	0.56 to 1.31	0.471	0.76	0.46 to 1.23	0.257
Females							
Antibody (anti‐SEA) negative	95/298 (32)	Ref	–	–	Ref	–	–
Antibody (anti‐SEA) positive	33/96 (34)	1.12	0.69 to 1.82	0.650	1.04	0.62 to 1.74	0.891

FSW cohort	HIV SC/Total (%)	IRR	95% CI	*p*	aIRR^c^	95% CI	*p*
Active or prior schistosome infection							
Females							
Antibody (anti‐SEA) negative	205/853 (24)	Ref	–	–	Ref	–	–
Antibody (anti‐SEA) positive	125/439 (28)	1.26	0.97 to 1.65	0.088	1.18	0.90 to 1.55	0.235

CAA, circulating anodic antigen; FSW, female sex worker; SEA, soluble egg antigen.

^a^ The serodiscordant couples cohorts were adjusted for age, sex, and study/site combination. Male/female subgroup models did not adjust for sex;

^b^ schistosomiasis: samples with detectable antischistosomal antibodies (anti‐SEA) and schistosome antigens (CAA);

^c^ the FSW cohort was matched on year of study enrolment (two year bands) and adjusted for age and workplace.

### 3.3. Schistosome infection intensity and HIV‐1 acquisition risk

3.3

Since schistosome antigen levels correlate with worm burden and higher worm burden leads to increased schistosome ova and subsequent mucosal damage, we evaluated associations between schistosomiasis and HIV‐1 acquisition stratified by level of infection intensity (Table 3 and Table S1). Compared to individuals without evidence of prior schistosome infection, we found no evidence that individuals with high intensity infections faced an increased risk of HIV‐1 acquisition in the serodiscordant couples (aOR = 0.96, 95% CI 0.51 to 1.82) or FSW (aIRR = 0.91, 95% CI 0.58 to 1.43) cohorts. We also assessed if there was a linear trend of increasing HIV‐1 acquisition risk across increasing schistosome infection intensity levels, but did not find an association in either the serodiscordant couples (*p* = 0.917, females subgroup *p* = 0.331) or FSW cohorts (*p* = 0.796).

**Table 3:** Associations between schistosomiasis infection intensity and the risk of HIV‐1 acquisition

Serodiscordant couples cohorts	Male and female				Female				
	HIV SC/total (%)	aOR^a^	95% CI	*p*	HIV SC/total (%)	aOR^a^	95% CI	*p*
No infection^b^	172/653 (26)	Ref	–	–	95/298 (32)	Ref	–	–
Past but not current	21/111 (19)	0.66	0.39 to 1.12	0.127	12/41 (29)	0.86	0.41 to 1.80	0.681
Low intensity	14/54 (26)	1.00	0.52 to 1.94	0.996	4/15 (27)	0.69	0.23 to 2.08	0.506
Moderate intensity	21/75 (28)	1.07	0.61 to 1.89	0.816	9/23 (39)	1.22	0.48 to 3.06	0.676
High intensity	17/65 (26)	0.96	0.51 to 1.82	0.901	8/17 (47)	1.82	0.59 to 5.61	0.294

FSW cohort	HIV SC/total (%)	aIRR^c^	95% CI	*p*	HIV SC/total (%)	aIRR^c^	95% CI	*p*
No infection^b^	–	–	–	–	205/853 (24)	Ref	–	–
Past but not current	–	–	–	–	43/149 (29)	1.24	0.84 to 1.83	0.280
Low intensity	–	–	–	–	20/61 (33)	1.47	0.83 to 2.63	0.188
Moderate intensity	–	–	–	–	32/111 (29)	1.28	0.80 to 2.07	0.305
High intensity	–	–	–	–	30/118 (25)	0.91	0.58 to 1.43	0.684

CAA, circulating anodic antigen; FSW, female sex worker; SEA, soluble egg antigen.

^a^ The serodiscordant couples cohorts were adjusted for age, sex and study/site combination. Female subgroup models did not adjust for sex;

^b^ definition of infection intensity categories: No infection (anti‐SEA negative), past infection (anti‐SEA positive & CAA < 10 pg/mL), low intensity (anti‐SEA positive & CAA 10 to 99 pg/mL), medium intensity (anti‐SEA positive & CAA 100 to 999 pg/mL) and high intensity (anti‐SEA positive & CAA ≥ 1000 pg/mL);

^c^ the FSW cohort was matched on year of study enrolment (two‐year bands) and adjusted for age and workplace.

### 3.4 Species‐specific schistosome infection and HIV‐1 acquisition risk

Lastly, we evaluated if specific schistosome species were associated with HIV‐1 acquisition risk (Table 4 and Table S2). In the serodiscordant couples cohorts, there was a 4% (36/957) prevalence of *S. haematobium* and 14% (134/957) prevalence of *S. mansoni*. In the FSW cohort the prevalence of *S. haematobium* and *S. mansoni* were 6% (71/1290) and 18% (233/1290) respectively. In the serodiscordant couples cohorts we found no evidence that either *S. mansoni* (aOR = 0.90, 95% CI 0.56 to 1.44) or *S. haematobium* (aOR = 1.06, 95% CI 0.46 to 2.40) were associated with HIV‐1 acquisition risk. Restricting to female participants, we still identified no association. Similarly, we did not find a statistically significant association between *S. mansoni* (aIRR = 0.83, 95% CI 0.58 to 1.20) or *S. haematobium* (aIRR = 1.64, 95% CI 0.93 to 2.87) and HIV‐1 acquisition risk in the FSW cohort. Additionally, we stratified positive results for each schistosome species by level of infection intensity and found no evidence that individuals with high intensity infections caused by *S. mansoni* or *S. haematobium* experienced increased HIV‐1 acquisition risk.

**Table 4:** Schistosome species‐specific associations with the risk of HIV‐1 acquisition

Serodiscordant couples cohorts^a^	Male and female					Female			
	HIV SC/total (%)	aOR^b^	95% CI	*p*	HIV SC/total (%)		aOR^b^	95% CI	*p*
Species‐specific associations with HIV‐1 acquisition risk^c^									
No active infection	193/764 (25)	Ref	–	–	107/339 (32)		Ref	–	–
*Schistosoma mansoni*	31/134 (23)	0.90	0.56 to 1.44	0.660	13/36 (36)		1.23	0.56 to 2.68	0.611
*Schistosoma haematobium*	10/36 (28)	1.06	0.46 to 2.40	0.898	2/9 (22)		0.44	0.08 to 2.29	0.328
Undetermined species	15/42 (36)	1.51	0.76 to 3.02	0.238	6/13 (46)		1.47	0.45 to 4.81	0.523
*S. mansoni* infection intensity and HIV‐1 acquisition risk^d^									
No active *S. mansoni* infection	214/823 (26)	Ref	–	–	115/357 (32)		Ref	–	–
Low intensity infection	7/34 (21)	0.76	0.32 to 1.83	0.546	2/9 (22)		0.53	0.12 to 2.29	0.394
Moderate intensity infection	14/53 (26)	1.07	0.55 to 2.07	0.846	5/14 (36)		1.02	0.31 to 3.33	0.973
High intensity infection	10/47 (21)	0.76	0.36 to 1.61	0.470	6/13 (46)		1.95	0.54 to 7.08	0.312
*S. haematobium* infection intensity and HIV‐1 acquisition risk^d^									
No active *S. haematobium* infection	235/921 (26)	Ref	–	–	126/384 (33)		Ref	–	–
Low intensity infection	2/7 (29)	0.97	0.16 to 5.78	0.975	0/2 (0)		–	–	–
Moderate intensity infection	5/16 (31)	1.18	0.38 to 3.62	0.778	1/4 (25)		0.51	0.06 to 4.57	0.548
High intensity infection	3/13 (23)	0.77	0.19 to 3.07	0.707	1/3 (33)		0.87	0.11 to 6.74	0.892

FSW cohort^a^	HIV SC/total (%)	aIRR^b^	95% CI	*p*	HIV SC/total (%)	aIRR^b^	95% CI	*p*
Species‐specific associations with HIV‐1 acquisition risk^c^								
No active infection	–	–	–	–	248/997 (25)	Ref	–	–
*S. mansoni*	–	–	–	–	57/232 (25)	0.83	0.58 to 1.20	0.326
*S. haematobium*	–	–	–	–	25/70 (36)	1.64	0.93 to 2.87	0.087
Undetermined species	–	–	–	–	11/26 (42)	1.89	0.88 to 4.08	0.104
*S. mansoni* infection intensity and HIV‐1 acquisition risk^d^								
No active *S. mansoni* infection	–	–	–	–	271/1052 (26)	Ref	–	–
Low intensity infection	–	–	–	–	10/40 (25)	0.95	0.45 to 2.02	0.899
Moderate intensity infection	–	–	–	–	23/88 (26)	1.09	0.63 to 1.88	0.767
High intensity infection	–	–	–	–	24/104 (23)	0.75	0.47 to 1.22	0.248
*S. haematobium* infection intensity and HIV‐1 acquisition risk^d^								
No active *S. haematobium* infection	–	–	–	–	303/1214 (25)	Ref	–	–
Low intensity infection	–	–	–	–	6/19 (32)	1.28	0.45 to 3.61	0.640
Moderate intensity infection	–	–	–	–	10/25 (40)	1.90	0.78 to 4.60	0.157
High intensity infection	–	–	–	–	9/26 (35)	1.30	0.55 to 3.10	0.549

CAA, circulating anodic antigen; FSW, female sex worker; SEA, soluble egg antigen.

^a^ Due to insufficient sample volumes, 3 samples were excluded from species testing: serodiscordant couples cohorts (n = 1) and FSW cohort (n = 2). In the FSW cohort, both samples were seroconverters, leading to the exclusion of their matched controls (n = 6). Additionally, some individuals were co‐infected with both schistosome species: 19 individuals (4 seroconverters) in the serodiscordant couples cohorts and 41 individuals (13 seroconverters) in the FSW cohort

^b^ the serodiscordant couples cohorts were adjusted for age, sex and study/site combination. Female subgroup models did not adjust for sex. The FSW cohort was matched on year of study enrolment (two‐year bands) and adjusted for age and workplace

^c^ definition of species‐specific categories: No active infection (anti‐SEA negative or CAA < 10 pg/mL), *S. mansoni* infection (anti‐SEA positive, CAA ≥ 10 pg/mL, and *S. mansoni* immunoblot positive), *S. haematobium* infection (anti‐SEA positive, CAA ≥ 10 pg/mL, and *S. haematobium* immunoblot positive) and undetermined species (anti‐SEA positive, CAA ≥ 10 pg/mL, and both *S. haematobium* and *S. mansoni* immunoblot negative)

^d^ definition of infection intensity categories: No active infection (anti‐SEA negative or CAA < 10 pg/mL or species immunoblot negative), low intensity (CAA 10 to 99 pg/mL), medium intensity (CAA 100 to 999 pg/mL) and high intensity (CAA ≥ 1000 pg/mL).

## 4 Discussion

In this analysis from four large prospective cohort studies from East Africa, we did not find a statistically significant association between schistosomiasis and the risk of HIV‐1 acquisition. In both the serodiscordant couples and FSW cohorts, the majority of schistosomiasis was caused by *S. mansoni* infection. The lack of an association between *S. mansoni* and HIV‐1 acquisition risk was consistent as we explored the data across multiple subgroups: FSW, serodiscordant couples, male, females and by levels of infection intensity. These results suggest that *S. mansoni* is not a major driver of the HIV‐1 epidemic throughout sub‐Saharan Africa.

We did not identify a statistically significant association between *S. haematobium* and HIV‐1 acquisition risk, overall or in sex‐stratified analyses. One hypothesis in the field has been that *S. haematobium* increases HIV‐1 acquisition risk specifically for women. In our analyses, the point estimate for HIV‐1 acquisition risk among FSWs with *S. haematobium* was 1.64, but this was not statistically significant. Our statistical power was limited with only 70 *S. haematobium*‐infected individuals in the cohort. Our results across all subgroups suggest that schistosomiasis is not associated with a large increased risk of HIV‐1 acquisition (RR ≥ 3), as suggested by some studies [5, 7, 9]; this finding is consistent with another recent study which found a moderate positive association between *S. haematobium* and HIV (aHR = 1.4) [12]. Very large studies would be needed to have sufficient statistical power to rule out whether *S. haematobium* is associated with a more moderate increase in HIV‐1 acquisition risk among women.

Little evidence suggests schistosomiasis increases HIV‐1 acquisition risk among men. Three of the four original cross‐sectional studies that found an association between schistosomiasis and HIV‐1 included only female participants [5, 6, 7, 8]. A recent longitudinal analysis found an association between *S. mansoni* and HIV‐1 acquisition, but it was specific to women, with no association observed among men [9]. Most cross‐sectional studies that included both sexes found no evidence of an association [13, 48, 49, 50], as did a recent study which included only men [51]. Our results are consistent with these findings.

Though the existing evidence suggesting an association between schistosomiasis and HIV‐1 acquisition among women is stronger than among men [5, 6, 7], findings have been mixed for both *S. mansoni* and *S. haematobium* [13, 52]. Our results are not consistent with a recent study that found *S. mansoni* infection associated with increased HIV‐1 acquisition risk among women in Tanzania [9]. A challenge when evaluating the overall strength of existing evidence is that many published analyses did not present separate subgroup analyses for males and females. This was the case for two recent longitudinal studies [10, 11] and previously published cross‐sectional studies that found no association between schistosomiasis and HIV‐1 acquisition [48, 49, 50]; the proportion of female participants in these study populations varied from 28% to 75%. Although the existing epidemiological evidence suggesting associations between HIV‐1 acquisition risk and schistosomiasis is inconsistent for both schistosome species, evidence for a biological mechanism is much stronger for *S. haematobium*. *S. haematobium* ova‐induced genital damage has been well documented, and genital bleeding and blood in urine are commonly observed symptoms of individuals infected by *S. haematobium* [53, 54, 55].

Some researchers who observed associations between schistosomiasis and HIV‐1 acquisition had intentionally selected a study population with very high schistosome infection burdens [6], such as communities living adjacent to schistosome‐infected bodies of water [7]. In contrast, participants in the present studies were selected because of their elevated risk of HIV‐1 acquisition and were enrolled at 10 locations across Kenya and Uganda. The prevalence of schistosomiasis in our study populations was similar to the estimated national prevalence for Kenya and Uganda, 23% and 20% respectively [56], suggesting that our findings should be generalizable to the general populations of these countries and much of sub‐Saharan Africa. Additionally, we found no evidence of increased HIV‐1 acquisition risk among individuals with high intensity infections, who would be expected to have the most schistosome ova‐induced genital damage.

Strengths of our analysis include the use of two distinct high‐risk populations. The serodiscordant couples and FSW cohorts each independently included more HIV‐1 seroconverters than any prior longitudinal analysis estimating the association between schistosomiasis and HIV. Thus, this manuscript includes the two best‐powered analyses conducted to‐date that provide valid estimates of association. The large amount of demographic, behavioural and clinical information collected from study participants enabled thorough adjustment for potential confounders. The schistosomiasis testing algorithm differentiated between active and previous schistosome infections. This permitted us to conduct secondary analyses evaluating if individuals with active or previous schistosome infections faced increased the risk of HIV‐1 acquisition, addressing the possibility that damage from schistosome ova and increased HIV‐1 susceptibility persist beyond the period of active infection [46]. Additionally, the antigen results allowed us to perform sub‐analyses by schistosome infection intensity, while the species‐specific immunoblots enabled us to evaluate associations for each schistosome species.

Since we utilized data from previously conducted cohort studies, one limitation is that we lacked data on clinical manifestations of schistosome infection among our study population. Through genital examinations, damage from schistosome ova can be identified most specifically as “grainy sandy patches” [53], and it is possible that only women with these patches have increased HIV‐1 acquisition risk. However, all past analyses that observed associations between schistosomiasis and HIV‐1 relied on laboratory diagnostic testing to identify their schistosome‐infected populations. The single cross‐sectional study that evaluated associations between grainy sandy patches and HIV‐1 did not find a statistically significant association [6]. Additionally, to address this limitation, we performed sub‐analyses by level of infection intensity, since participants with high‐intensity infections might be expected to have the most severe genital damage from schistosome ova. A second limitation is that though the SCAA20 assay used to identify active infections has a high sensitivity (80% to 95%), it lacks the sensitivity to detect very low‐burden infections. However, three of the six studies that found an association between schistosomiasis and HIV‐1 relied on either the SCAA20 [7], SEA ELISA [8], or species‐specific immunoblots [12] we utilized, and we expect that any individuals with low‐burden infections misclassified by our assay would have limited schistosome ova‐induced genital damage. A final limitation is that we lacked data on praziquantel treatment among participants in the four cohorts. However, the fact that in both study populations approximately two out of three individuals with antis chistosomal antibodies had active infections (a positive antigen result) suggests that praziquantel treatment was infrequent in these populations.

## 5 Conclusions

We found no statistically significant effect of schistosome infection on HIV‐1 acquisition among this diverse population from East Africa. Our evidence was robust that *S. mansoni* was not associated with an increased risk of HIV‐1 acquisition, in men or women. In the FSW cohort, but not the serodiscordant couples cohorts, *S. haematobium* was associated with a point estimate of elevated HIV‐1 risk but this was not statistically significant and there was no dose‐response effect. Regardless of whether or not schistosomiasis impacts HIV‐1 acquisition risk, reducing the morbidity and mortality caused by schistosome infections necessitates the continued expansion of preventive treatment for schistosomiasis [57].

## Competing interests

RSM has received funding for research, paid to the University of Washington, from Hologic Corporation. All other authors declare that they have no competing interests relevant to this work.

## Authors’ contributions

AFB, JMB, CC and RVB initially conceived of the study, with WES, GJD, PLAMC, AAS, SMN, NRM and RSM contributing to the study design. AFB, JMB, WES, GJD, PLAMC, AN, NRM, CC, AM, RSM and RVB supported acquiring and interpreting the data, while AFB, JMB, ASS and RVB were involved in the analysis. The manuscript was prepared by AFB, and all authors contributed to revision of the manuscript and approved the final version.

## Supporting information


**Table S1.** Associations between schistosomiasis infection intensity and the risk of HIV‐1 acquisition among men
**Table S2.** Schistosome species‐specific associations with the risk of HIV‐1 acquisition among men
