# Supplementary material for: Associations between schistosomiasis and HIV‐1 acquisition risk in four prospective cohorts: a nested case‐control analysis
Source: J Int AIDS Soc. 2020 Jun 25;23(6):e25534. doi: 10.1002/jia2.25534 (PMC7316390; doi:10.1002/jia2.25534)
Supplement: Supplementary file 1 — Table S1. Associations between schistosomiasis infection intensity and the risk of HIV‐1 acquisition among men Table S2. Schistosome species‐specific associations with the risk of HIV‐1 acquisition among men [file JIA2-23-e25534-s001.docx]

**Supporting Information**

**Table S1 Associations between schistosomiasis infection intensity and the risk of HIV-1 acquisition among men**

|  | **Male** | | | |
| --- | --- | --- | --- | --- |
| **Serodiscordant couples cohorts** | **HIV SC/Total (%)** | **aOR^2^** | **95% CI** | ***P*** |
| No evidence of infection^1^ | 77/355 (22) | Ref | — | — |
|  |  |  |  |  |
| Past infection | 9/70 (13) | 0.51 | 0.23-1.13 | 0.098 |
| Low intensity | 10/39 (26) | 1.10 | 0.49-2.44 | 0.823 |
| Moderate intensity | 12/52 (23) | 0.92 | 0.43-1.97 | 0.839 |
| High intensity | 9/48 (19) | 0.70 | 0.30-1.68 | 0.430 |

^1^ Adjusted for age, and study/site combination.

^2^ Definition of infection intensity categories: No evidence of infection (anti-SEA negative), past infection (anti-SEA positive & CAA <10 pg/ml), low intensity (anti-SEA positive & CAA 10-99 pg/ml), medium intensity (anti-SEA positive & CAA 100-999 pg/ml), and high intensity (anti-SEA positive & CAA ≥1000 pg/ml).

**Table S2 Schistosome species-specific associations with the risk of HIV-1 acquisition among men**

|  | | **Male** | | | | | |
| --- | --- | --- | --- | --- | --- | --- | --- |
| **Serodiscordant couples cohorts** | **HIV SC/Total (%)** | | **aOR^1^** | **95% CI** | ***P*** |  |  |
| Species-specific associations with HIV-1 acquisition risk^2^ | | | | | | |  |
| No active infection | 86/425 (20) | | Ref | — | — |  |  |
| *S. mansoni* | 18/98 (18) | | 0.73 | 0.39-1.36 | 0.317 |  |  |
| *S. haematobium* | 8/27 (30) | | 1.47 | 0.54-4.00 | 0.454 |  |  |
| Undetermined species | 9/29 (31) | | 1.58 | 0.68-3.67 | 0.291 |  |  |
| *S. mansoni* infection intensity and HIV-1 acquisition risk^3^ | | | | | | |  |
| No active *S. mansoni* infection | 99/466 (21) | | Ref | — | — |  |  |
| Low intensity infection | 5/25 (20) | | 0.83 | 0.28-2.44 | 0.739 |  |  |
| Moderate intensity infection | 9/39 (23) | | 1.01 | 0.44-2.31 | 0.980 |  |  |
| High intensity infection | 4/34 (12) | | 0.42 | 0.13-1.29 | 0.130 |  |  |
| *S. haematobium* infection intensity and HIV-1 acquisition risk^3^ | | | | | | |  |
| No active *S. haematobium* infection | 109/537 (20) | | Ref | — | — |  |  |
| Low intensity infection | 2/5 (40) | | 1.79 | 0.27-11.9 | 0.547 |  |  |
| Moderate intensity infection | 4/12 (33) | | 1.43 | 0.41-5.00 | 0.578 |  |  |
| High intensity infection | 2/10 (20) | | 0.81 | 0.12-5.58 | 0.834 |  |  |

^1^ Adjusted for age and study/site combination.

^2^ Definition of species-specific categories: No active infection (anti-SEA negative or CAA <10 pg/ml), *S. mansoni* infection (anti-SEA positive, CAA ≥10 pg/ml, and *S. mansoni* immunoblot positive), *S. haematobium* infection (anti-SEA positive, CAA ≥10 pg/ml, and *S. haematobium* immunoblot positive) and undetermined species (anti-SEA positive, CAA ≥10 pg/ml, and both *S. haematobium* and *S. mansoni* immunoblot negative).

^3^ Definition of infection intensity categories: No active infection (anti-SEA negative or CAA <10 pg/ml or species immunoblot negative), low intensity (CAA 10-99 pg/ml), medium intensity (CAA 100-999 pg/ml), and high intensity (CAA ≥1000 pg/ml).
